# Next-Generation Precision Breeding in Peanut (*Arachis hypogaea* L.) for Disease and Pest Resistance: From Multi-Omics to AI-Driven Innovations

**DOI:** 10.3390/insects17010063

**Published:** 2026-01-04

**Authors:** Xue Pei, Jinhui Xie, Chunhao Liang, Aleksandra O. Utkina

**Affiliations:** 1 Institute of Plant Protection, Liaoning Academy of Agricultural Sciences, Shenyang 110161, China; 2 Institute of Environmental Engineering, RUDN University, 6 Miklukho-Maklaya St., Moscow 117198, Russia

**Keywords:** precision breeding, peanut, multi-omics, genome editing, artificial intelligence, phenomics, remote sensing, climate resilience, crop improvement, plant immunity, host-pathogen interactions

## Abstract

Peanut (*Arachis hypogaea* L.) is a globally important oilseed and food legume, yet its productivity is persistently constrained by devastating diseases and insect pests that thrive under changing climates. This review aims to provide a comprehensive synthesis of advances in precision breeding and molecular approaches for enhancing disease and pest resistance in peanut. Traditional control measures ranging from crop rotation and cultural practices to chemical protection have delivered only partial and often unsustainable relief. The narrow genetic base of cultivated peanut and its complex allotetraploid genome further hinder the introgression of durable resistance. Recent advances in precision breeding are redefining the possibilities for resilient peanut improvement. Multi-omics platforms genomics, transcriptomics, proteomics, and metabolomics have accelerated the identification of resistance loci, effector-triggered immune components, and molecular cross-talk between pathogen, pest, and host responses. Genome editing tools such as CRISPR-Cas systems now enable the precise modification of susceptibility genes and defense regulators, overcoming barriers of conventional breeding. Integration of these molecular innovations with phenomics, machine learning, and remote sensing has transformed resistance screening from manual assessment to real-time, data-driven prediction. Such AI-assisted breeding pipelines promise enhanced selection accuracy and faster deployment of multi-stress-tolerant cultivars. This review outlines current progress, technological frontiers, and persisting gaps in leveraging precision breeding for disease and pest resistance in peanut, outlining a roadmap toward climate-resilient, sustainable production systems.

## Simple Summary

Peanut is an important crop grown for food and oil around the world. However, its yield is often reduced by many diseases and insect pests, problems that are becoming worse due to climate change. Traditional methods like crop rotation, field management, and pesticide use have not been fully effective or sustainable. Because cultivated peanut has a narrow genetic base and a complex genome, developing resistant varieties through conventional breeding is difficult. New scientific tools are now helping overcome these limits. Techniques such as genomics, transcriptomics, and metabolomics are helping scientists find genes that control resistance. Modern gene-editing methods like CRISPR-Cas allow precise changes to improve the plant’s natural defense. With the help of artificial intelligence, phenotyping, and remote sensing, resistance testing has become faster and more accurate. Together, these modern tools are helping breeders develop peanut varieties that can better resist diseases and pests, ensuring stable and sustainable production in the future.

## 1. Introduction

Peanut (*Arachis hypogea* L.), commonly known as groundnut, is a globally important legume and cash crop cultivated extensively across tropical and subtropical regions. Its high oil and protein content make it an essential source of edible oil, food, organic fertilizer and fodder [1]. In recent years, peanuts have gained additional prominence as a functional food, attributes to their abundance of bioactive compounds including phytosterols, phenolics, stilbenes, lignans, and isoflavonoid that exhibit potent antioxidant and health-promoting properties [2]. Peanuts are consumed globally in diverse forms; raw, boiled, roasted, or processed into oil, paste, snacks, and confectionery products, which demonstrate their nutritional versatility and commercial significance [3]. From an agronomic perspective, peanut cultivation continues to expand, primarily in Asia and Africa which together account for more than 90% of the total global acreage [4]. China remains the world’s largest producer, contributing approximately of 18.36 million tons (MT) annually, while India holds the largest cultivation area [5]. Continuous improvement in agricultural policies, mechanization, and crop management has led to steady gains in productivity [6]. However, these gains are increasingly threatened by escalating biotic stresses, particularly diseases and insect pests that significantly constrain yield potential and product quality [7].

Peanut production is challenged by a broad spectrum of biotic agents, including more than 350 insect species, of which *Aphis craccivora*, and root-knot nematodes (*Meloidogyne arenaria*) poses a global threat to peanut-growing areas [8,9]. Other pests such as *Helicoverpa armigera* and *Spodoptera litura* are more regionally significant: *H. armigera* is widely distributed across Africa, Asia, Australia and Europe, and has recently established in South America. The distribution of *S. litura* likewise spans much of Asia and parts of North Africa, Australia and Oceania [10,11]. These pests inflict severe damage to both aerial and subterranean plant parts, leading to stunted growth, impaired photosynthetic capacity, disrupted root physiology, and substantial yield loss [12]. Complementing this, major fungal diseases such as early and late leaf spots, rust, stem rot, and *Aspergillus flavus* infection continue to impose heavy economic losses globally [13]. The coexistence of diverse pathogens and pests in the same agroecosystem creates overlapping stress networks that complicate management strategies and threaten production sustainability under changing climatic conditions,

Despite decades of research, diseases and pests remain persistent constraints in peanut production. Traditional control strategies including crop rotation, chemical protection, and cultural management have achieved partial success [14,15,16]. However, their scalability and consistency are limited by environmental variability, rising input costs, and ecological concerns. Chemical pesticides, though effective in the short term, are increasingly linked to pesticide resistance, biodiversity loss, and food safety issues. Consequently, there is a growing need for sustainable and precise disease and pest management approaches capable of addressing dynamic pathogen populations and complex field interactions with reduced environmental footprint [17,18,19].

To achieve long-term resilience, peanut improvement programs have progressively shifted toward integrating molecular and precision breeding approaches that complement traditional agronomy. These include genomics-assisted breeding, genome-wide selection, multi-omics analysis, gene editing tools such as Clustered Regularly Interspaced Short Palindromic Repeats and CRISPR-associated protein 9 (CRISPR/Cas systems), and speed breeding platforms, which collectively enable targeted enhancement of pest and disease resistance [20]. By leveraging genomic insights into host-pathogen interactions and the genetic architecture of resistance traits, these technologies promise durable and climate-resilient cultivars that reduce dependency on chemical interventions and sustain yield stability in variable environments.

Over the past decade, several reviews have provided valuable perspectives on discrete aspects of peanuts research ranging from traditional disease management [14,21], nano-fertilizer applications [22], and somatic embryogenesis to medicinal and functional attributes, and improved cultural practices for organic cultivation [23]. However, a major knowledge gap remains in connecting molecular-scale innovations with field-oriented pest and disease management frameworks. Most existing literature treats genomic and breeding advancements in isolation, lacking a comprehensive systems perspective that integrates multi-omics-driven discovery, genome editing precision, and digital phenotyping analytics within an applied pest and pathogen management context.

This review addresses that critical gap by offering an integrative synthesis of traditional and precision breeding strategies for managing diseases and insect pests in *Arachis hypogaea*. Specifically, it elucidates the molecular and physiological underpinnings of peanut-pathogen-insect interactions, advances in genomic and phenomics tools for resistance breeding, and the role of artificial intelligence, remote sensing, and predictive modelling in next-generation crop protection. The novelty of this review lies in bridging the continuum from laboratory discovery to field deployment highlighting how precision breeding and digital technologies can together enable climate-smart, durable resistance against evolving pest and pathogen threats. By integrating multi-omics, genome editing, and real-time phenotyping, this review aims to chart a systems-level roadmap toward sustainable peanut health management and accelerated resistance breeding in the era of precision agriculture.

## 2. Current Understanding of Peanut–Pathogen–Pest Interactions

Peanut is constantly subjected to multifaceted biotic stresses, including pathogens and insect pests that compromise both yield and quality. The interaction between peanut and its biotic adversaries operates across complex molecular, biochemical, and ecological layers. A comprehensive understanding of these interactions is fundamental to designing next-generation breeding programs that exploit host defense networks and enhance resilience against evolving pathogen-pest pressures.

### 2.1. Major Diseases and Their Impact

#### 2.1.1. Early and Late Leaf Spot (*Cercospora arachidicola*, *C. personatum*)

Among foliar diseases, early leaf spot (ELS), caused by *Passalora arachidicola* (Syn. *Cercospora arachidicola*), and late leaf spot (LLS) caused by *Nothopassalora personata* (Syn. *Cercosporidium personatum*), remain the most destructive and widely distributed in peanut-growing regions. These Ascomycete fungal initiate infection on foliage, forming small necrotic lesions that expand, coalesce, and cause premature defoliation (Table 1). Defoliation substantially diminishes photosynthetic efficiency, leading to yield reductions of up to 70%. The disease cycle is perpetuated by conidial dispersal through wind, allowing rapid field-to-field transmission [24].

#### 2.1.2. Rust (*Puccinia arachidis*)

Peanut rust, caused by *P. arachidis* Speg., represents another globally significant foliar disease within the Basidiomycota lineage [25] (Table 1). First reported from Suriname in 1827 and later recognized as a distinct species by Spegazzini in 1976, the pathogen now occurs in nearly all major peanut-producing regions. Its epidemiology is favoured by warm, humid environments that support rapid uredospore cycling, enabling explosive spread during conducive conditions [26]. Rust infection leads to significant physiological impairment, manifesting as early pod maturity, smaller seeds, reduced oil content, and deteriorated haulm quality [27]. In susceptible cultivars such as Robut 33-1, yield reductions of up to 57% have been recorded. While screening efforts have identified eight rust-resistant accessions among 700 genotypes from Peru [28], and several wild *Arachis* relatives exhibits high resistance [29], the genetic basis of durable resistance remains an active area of investigation. Such wild germplasm serves as an invaluable reservoir for resistance gene introgression through precision breeding and genomic-assisted breeding.

#### 2.1.3. Aflatoxin Contamination (*Aspergillus flavus*, *A. parasiticus*)

Aflatoxins, highly toxic polyketide-derived secondary metabolites primarily produced by *Aspergillus flavus* and *A. parasiticus*, are among the most serious food safety concerns associated with peanuts [30,31]. The major aflatoxin analogues: AFB_1_, AFB_2_, AFG_1_, AFG_2_, AFM_1_, and AFM_2_ exhibit strong carcinogenic potential, with AFB_1_ recognized as a potent hepatocarcinogen that induces TP53 gene mutations. Chronic dietary exposure leads to impaired growth, micronutrient deficiencies, and immune suppression in human [32], while contaminated feed causes productivity losses and toxin carryover into milk and animal products [33]. Infection by *Aspergillus* spp. can occur at pre-harvest, harvest and post-harvest durations, particularly in regions characterized by heat and drought stress [34]. Environmental factors such as high temperature, limited moisture, and insect-mediated wounding predispose peanut pods and kernels to fungal invasion and subsequent toxin biosynthesis [35]. The complexity of aflatoxin contamination lies in its multifactorial nature linking pathogen ecology, host physiology, and abiotic stress, necessitating integrated interventions spanning genomics, biocontrol, and post-harvest management.

#### 2.1.4. Bacterial Wilt (*Ralstonia solanacearum*)

Bacterial wilt (BW), caused by *Ralstonia solanacearum*, has emerged as a severe soilborne disease, particularly in China and Southeast Asia [36] (Table 1). The disease currently affects over one million hectares in China, representing nearly 18% of the national peanut cultivation area. Yield losses range from 10–30%, with localized epidemics reaching beyond 50 per cent [37]. In addition to direct yield suppression, *R. solanacearum* infection predisposes plants to aflatoxin contamination by altering pod and kernel integrity [38]. Breeding for resistance remains the most sustainable management approach. More than 20 resistant peanut cultivars have been developed, effectively reducing wilt incidence to below 7% in epidemic regions [39]. Nevertheless, the productivity of resistant cultivars often lags behind susceptible high-yielding types, posing a key challenge for breeders [40]. Future progress depends on coupling resistance breeding with yield-enhancing traits through genomic selection and molecular introgression to achieve both resilience and productivity in BW-prone areas.

#### 2.1.5. The Root-Knot Nematode (RKN) (*Meloidogyne arenaria*)

Among nematode pests, *Meloidogyne arenaria*, the root-knot nematode is recognized as a major biotic constraint in peanut production worldwide [41]. Yield reductions of approximately 12% have been reported in heavily infested Chinese fields [42]. RKNs are obligate endoparasites capable of infecting most commercial peanut varieties [43]. Their life cycle begins with second-stage juveniles (J2s) penetrating root tips and establishing feeding sites in the vascular differentiation zone. As feeding progresses, giant cell formation and galling disrupt nutrient and water translocation, manifesting as stunting, chlorosis, and patchy field symptoms. Under hot, dry conditions, RKN stress can induce early senescence and plant death [44]. The systemic physiological dysfunction limits the plant’s ability to sustain growth, often leading to severe yield penalties even under normal soil fertility. Furthermore, galled roots provide entry points for secondary pathogens, compounding losses through complex disease syndromes. Addressing RKN requires integrated management combining host resistance, biological control, and precision soil diagnostics to sustain peanut productivity in nematode-endemic environments.

**Table 1:** Major Pathogenic Diseases Limiting Peanut (*Arachis hypogaea* L.) Productivity and Quality.

Types	Pathogen	Diseases	Symptoms	References
Bacterial	*Ralstonia solanacearum*	Bacterial wilt	Rapid wilting of the plant leading to eventual death.	[45,46]
Fungal	*Nothopassalora personata*	Late leaf spot (LLS)	Dark brown to black lesions on leaves, rapid defoliation	[14]
	*Cercospora arachidicola*	Early leaf spot (ELS)/Cercospora leaf spot (CLS)	Causes circular or irregular leaf lesions surrounded by yellow halos	[47]
	*Pythium myriotylum*/*Rhizoctonia solani*	Pod rot	Pods become soft, mushy, or shrivelled with discoloured kernels, leading to pod rot and damaged peanuts.	[48]
	*Aspergillus flavus*/*Aspergillus parasiticus*	Aflatoxin contamination	Moldy seeds/potential aflatoxin contamination	[49,50]
	*Scelrotium rolfsii*	Stem rot	Mycelium covers the stem near the soil surface, with sclerotia formation on diseased tissue.	[51,52]
Viral	Groundnut rosette assistor virus (GRAV)	Groundnut rosette disease (GRD)	Mosaic patterns or necrotic lesions on leaves, severe stunting, shortened internodes, and reduced leaf size.	[53]
	Groundnut bud necrosis virus	Peanut stem necrosis disease (PSND)	Necrotic spots and streaks on stem, petiole, and buds, chlorotic mottling on leaves, stunting, and proliferation of axillary shoots.	[54,55]

### 2.2. Major Insect Pests of Peanut: Dynamics, Damage, and Global Impact

Peanut is a vital oilseed and food legume cultivated worldwide, yet its productivity is persistently constrained by an array of insect pests that attack the crop from seedling establishment through post-harvest storage (Table 2). Insect infestation not only reduced yield but also undermines oil content, seed quality, and food safety posing a major challenge for sustainable peanut production systems [56]. Globally, more than 360 insect species have been documented to infest groundnut at various growth and storage stages [57], underscoring the crop’s vulnerability to complex pest assemblages that operate across spatial and temporal scales.

#### 2.2.1. Sucking Pests and Defoliators

Above-ground insect pests constitute the first and often visible line of attack against peanut plants. Among them, sap-sucking insects including leaf hoppers (*Empoasca kerri*), aphids (*Aphis craccivora*), thrips (*Scirtothrip dorsalis*, *Frankliniella schultzei*, *Frankliniella fusca*), spittle bug (*Locris* sp.), and stink bug (*Nezara virudula*) cause direct physiological stress by extracting phloem sap from tender leaves and shoots [58]. Their feeding disrupts chlorophyl biosynthesis and photosynthetic efficiency, resulting in chlorosis, leaf curling, stunted growth, and, ultimately, substantial yield losses. More critically, these insects act as efficient vectors for several viral pathogens, accelerating disease epidemics and compounding the biotic stress load on peanut crops [58].

The second major guild of foliar pests comprises leaf feeders and defoliators, including leaf miners (*Aproaerema modicella*), the red-headed hairy caterpillar (*Amsacta albistriga*), tobacco caterpillar (*S. litura*), African bollworm (*Heliothis* sp.), and gram pod borer (*H. armigera*) [58,59]. These insects feed aggressively on leaves, flowers, and young pods, reducing the photosynthetic surface area and disrupting reproductive success. In sub-Saharan Africa and parts of Asia, leaf miners have attained epidemic status, with severe infestations causing yield reductions and forcing farmers to adopt costly chemical interventions [59,60].

##### Groundnut Aphids (*Aphis craccivora*)

The groundnut aphid is one of the most economically significant pests with a cosmopolitan distribution and a remarkable capacity for host adaptation [61]. Aphid infestations cause both direct and indirect damage directly by removing phloem sap and introducing phytotoxic saliva, and indirectly by secreting honeydew that promotes sooty mold development and by serving as a vector for multiple viruses. It has been estimated that aphids can cause up to 20% yield loss in peanut, affecting plants from the seedling to the mature growth stages [61]. Notably, *A. craccivora* transmits at least seven economically important viruses, including Groundnut Rosette Virus (GRV) and Peanut Stripe Virus (PStV), which together can devastate entire fields in susceptible cultivars. Severe infestations result in yellowing, wilting, and premature death of plants, significantly compromising kernel yield and quality [62].

##### Groundnut Pod Borer (*Helicoverpa armigera*)

The polyphagous pod borer *H. armigera* ranks among the world’s most destructive agricultural pests, inflicting an estimated annual economic loss exceeding $2 billion globally [10]. It is prevalent across Africa, Asia, Australia, and Southern Europe and feeds on over 200 plant species, including peanut [63]. The larvae directly attack flowers, pods, and developing seeds, causing both quantitative and qualitative losses. In peanuts, larval feeding on reproductive structures leads to poor pod set, reduced kernel filling, and high post-harvest contamination [64]. The pest’s broad range, rapid reproductive potential, and resistance to multiple insecticide classes make it particularly difficult to control under traditional management regimes.

##### Spodoptera litura

*Spodoptera litura* represents another major foliar pest with devastating potential across tropical and subtropical peanut-growing regions. Its voracious larvae defoliate plants by consuming leaf lamina, leaving only midribs and severely impairing photosynthesis. Yield losses ranging from 35 to 55% have been recorded under severe infestations [65]. Maximum damage typically coincides with the flowering and pod formation stages critical periods determining final yield potential [11]. The pest’s polyphagous nature and capacity to develop resistance to conventional insecticides integrated approaches involving biological agents, pheromones traps, and resistant cultivars.

##### Root and Pod Feeders

While above-ground pests cause visible foliar damage, below-ground insect pests inflict more insidious yet equally devastating effects on peanut productivity. Root and pod feeders including termites (*Microtermes* sp., *Odontotermes* sp.), root mealybugs (*Parastictococcus multispinosus*), pod-sucking bugs (*Elasmolomus sordidus*), and white root grub (*Holotrichia serrata*, *Schizanycha* sp., *Schyzonycha fusca*, *Schyzomychz Africana*) attack peanut plants at multiple growth stages [66,67]. These pests feed on roots and pods, disrupting water and nutrient uptake, leading to wilting, stunting, and eventual plant death. The infestations, in particular, are often aggravated by drought conditions and poor organic matter content in soils, making them recurrent threats in semi-arid production systems.

Pod boring and soil-dwelling insects also predispose peanut to secondary fungal infections, especially by *Aspergillus flavus*, resulting in pre-harvest and post-harvest aflatoxin contamination a major food safety concern in peanut value chains [67]. Such interactions between insect injury and pathogenic exemplify the interconnectedness of pest and disease dynamics under field conditions.

#### 2.2.2. Storage Insect Pests: Hidden Post-Harvest Losses

Even after harvest, insect pests continue to undermine peanut quality storage and transport. Post-harvest losses contribute to an estimated 1.3 billion tons of global food wastage annually, with stored peanuts accounting for a significant share due to infestation by storage insects [68]. On average, 20% of stored commodities are lost annually worldwide due to insect damage and associated quality deterioration [69]. The primary storage pests belong to the orders Coleoptera, Lepidoptera, Hemiptera, and Psocoptera, including beetles (*Tribolium castaneum*, *Cryptolestes minutus*, and *Sitophilus oryza*), booklice (*Liposcelius decolor*), moths (*Ephestia cautella*, *Plodia interpunctella*, *Corcyra cephalonica*) [68]. These insects degrade seed quality by consuming endosperm, reducing germination potential, and contaminating stored produce with excreta and allergens. Infestations during storage not only diminish commercial value but also amplify the risk of aflatoxin build up under suboptimal storage conditions, linking field pest pressure to post-harvest food safety concerns.

The vast diversity and ecological plasticity of peanut insect pests from field to storage, underscore the necessity for integrated management frameworks that combine conventional tactics with molecular, ecological, and digital innovations. A nuanced understanding of pest-pathogen-host-interactions, alongside predictive modelling and resistance breeding, is imperative for mitigating yield and quality losses under changing climatic conditions. As the peanut industry transitions toward precision agriculture, addressing these pest complexities remains central to achieving sustainable crop resilience.

**Table 2:** Economically Important Insect Pests of Peanut (*Arachis hypogaea* L.) and Their Impact on Productivity and Seed Quality.

Insect Pests	Mode of Damage	References
Tobacco thrips (*Frankliniella fusca*)	Thrips feed on developing leaves and buds using rasng-sucking mouthparts, causing leaf discoloration, distortion, and stunting.	[70]
Groundnut bruchid (*Caryedon serratus*)	Larvae bore into seeds and feed on embryo and endosperm, creating exit holes and reducing seed weight, quality, and nutritive value.	[71]
Tobacco caterpillar (*S. litura*)	Larvae feed on leaf undersides, skeletonizing and destroying leaves, leaving only petioles and branches.	[72]
Cotton bollworm (*H. armigera*)	Larvae feed on leaf tissue, leaf edges, and flower buds, potentially destroying all new buds and severely reducing yield.	[73]
Black cutworm (*Agrotis ipsilon*)	Larvae cut tender stems and roots, killing plants, and burrow into pods to feed on kernels.	[73]
Groundnut leafminer (*Aproaerema modicella*)	Larvae feed on leaves, reducing photosynthetic area and lowering yield.	[74,75]
White grubs (*Holotrichia parallela*)	White grubs feed on roots, seeds, tubers, and pods underground, causing kernel and pod damage and reducing yield.	[76,77]
Groundnut sucking bug (*Rhyparochromus litotoralis*)	Bugs perforate pods and feed on seeds, causing shrivelling, increased free fatty acids, rancid flavour, and reduced kernel quality.	[78]
Groundnut pod borer (*Elasmolomus sordidus*)	Sucking activity deforms kernels, making them unfit for human and animal consumption.	[79]
Thrips (*Scirtothrips dorsalis*)	Transmit PYSV, causing yellow chlorotic spots and patches, leaf curling, necrosis, stunted growth, and eventual plant death.	[80,81]

## 3. Host Defense Mechanisms: Molecular, Biochemical, and Cellular Layers

**Figure 1:**
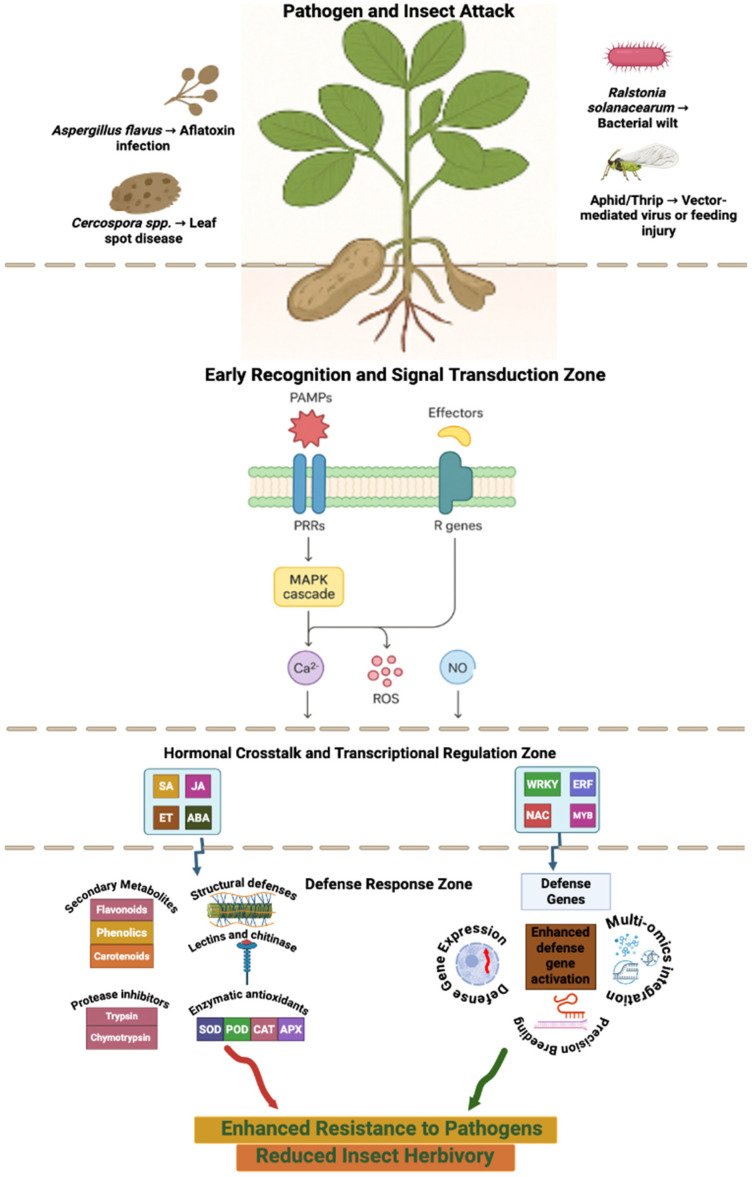
Schematic representation of molecular and biochemical defense pathways activated in peanut (*Arachis hypogaea* L.) against pathogens and insect pests. Upon attack, peanut cells recognize pathogen- or insect-derived molecules through pattern recognition receptors (PRRs) and resistance (R) proteins, initiating signal transduction cascades involving calcium influx, ROS generation, and MAPK activation. Hormonal cross-talk among salicylic acid, jasmonic acid, ethylene, and abscisic acid regulates transcription factors (WRKY, NAC, MYB, ERF) that drive expression of defense-related genes. The resulting biochemical responses include synthesis of PR proteins, antioxidants, phytoalexins, structural reinforcements, and anti-insect compounds that together enhance overall resistance.

Through millions of years of co-evolution, plants have developed a remarkably sophisticated and multi-layered immune system to withstand invasion by a broad range of pathogenic microorganisms. This intricate defense machinery integrates molecular perception, signal transduction, and metabolic reprogramming to maintain a delicate equilibrium between growth and immunity [82]. Once a plant perceives stress, either biotic or abiotic, a cascade of signalling events is triggered, engaging an interwoven network of morphological, physiological, and biochemical defense responses (Figure 1) [83].

### 3.1. Multi-Layered Plant Defense Architecture

Plants possess both passive and active defense strategies that collectively form a dynamic immune barrier. The passive or performed defenses consist of structural and chemical deterrents, including the waxy cuticle, lignified cell walls, and trichomes, which physically prevent microbial ingress and insect herbivory [84]. In groundnut, for example, seed coat thickness and reduced permeability confer significant resistance to *A. flavus* colonization, acting as a physical blockage against fungal penetration. The biochemical composition of the seed coat further enhances resistance, as protein profiling across fifteen peanut genotypes revealed elevated trypsin inhibitor in resistant lines compared with susceptible ones. Beyond these passive mechanisms, plants deploy a finely tuned innate immune system comprising two hierarchical yet interlinked layers: pattern-triggered immunity (PTI) and effector-triggered immunity (ETI) [85]. PTI relies on pattern-recognition receptors (PRRs) that sense pathogen-associated molecular patterns (PAMPs), thereby initiating an early defense response. However, successful pathogens often secrete virulence effectors to suppress PTI. In turn, plants have evolved intracellular nucleotide-binding site-leucine-rich repeat (NBS-LRR) receptors that recognize these effectors directly or indirectly, activating ETI; a more robust and specific immune response [86]. Notably, PTI and ETI are not isolated; instead, they synergistically reinforce each other to establish a comprehensive immune network crucial for durable resistance and crop improvement [87].

### 3.2. Early Signalling and Oxidative Dynamics

Upon pathogen recognition, one of the earliest host responses is the rapid production of reactive oxygen species (ROS) and the activation of mitogen-activate protein kinase (MAPK) cascades [88]. Concurrently, pest or pathogen attack triggers both local and systemic responses, often mediated by oligogalacturonides (OGAs), jasmonic acid (JA), and hydrogen peroxide (H_2_O_2_) signalling. In groundnut, *A. flavus* infection induces significant alterations in enzymatic activities related to oxidative metabolism and phenylpropanoid biosynthesis, such as phenylalanine ammonia lyase (PAL), peroxisome (POD), and lipoxygenase (LOX) [89,90]. These enzymes participate in lignin deposition, phenolic compound biosynthesis, and detoxification of ROS, all these processes are essential to maintain cellular integrity and limiting fungal spread [91].

### 3.3. Inducible Defense Proteins and PR Gene Families

An important downstream consequence of these defense cascades is the induction of a broad array of defense-related proteins, including proteinase inhibitors, lectins, chitinases, α-amylase inhibitors, and polyphenol oxidases. Among these, pathogenesis-related (PR) proteins represent one of the most studies groups in plant defense biology. PR proteins are encoded by genes that are transcriptionally activated under pathogen attack [92]. To date, at least 17 PR protein families have been identified, classified by their biochemical and molecular functions [93]. In peanuts, PR genes such as chitinase and PR10 have been implicated in conferring resistance to *A. flavus* and mitigating aflatoxin accumulation [94]. These inducible proteins form a key component of both the hypersensitive response (HR) and systemic acquired resistance (SAR), providing local containment and long-distance immune memory.

### 3.4. Hormonal Cross-Talk in Defense Regulation

Phytohormones orchestrate the complex regulatory network underlying plant immune responses (Figure 1). Among them, salicylic acid (SA), jasmonic acid (JA), and ethylene (ET) are central regulators linking PTI and ETI to downstream transcriptional reprogramming [95]. Typically, SA-mediated signalling activates defenses against biotrophic and hemi-biotrophic pathogens, whereas JA and ET pathways confer resistance to necrotrophic pathogens and chewing herbivores pathogens [96].

Experimental evidence underscores the functional significance of these hormonal networks in peanuts. Exogenous JA application enhances resistance to *S. litura* by increasing POD and PPO activates, as well as levels of phenolics, malondialdehyde, and hydrogen peroxide [97]. Similarly, transcriptomic analyses of *Arachis* genotypes resistant to root-knot nematodes revealed upregulation of genes associated with SA and JA signalling, as well as auxin regulation, emphasizing hormone-mediated defense cross-talk [98].

Beyond the canonical trio, several other hormones including abscisic acid (ABA), auxin, brassinosteroids (BRs), gibberellic acid (GA), cytokinin (CK), and peptide hormones contribute to the modulation of immune responses [99]. Among these, JA emerges as a pivotal integrator that interacts extensively with other hormonal pathways to fine-tune immune activation [100]. Despite significant advances, the molecular mechanisms governing peanut defense against insect pests and their interaction with disease resistance pathways remain poorly characterized. The intricate cross-talk between hormonal and defense signalling pathways offers both challenges and opportunities for precision breeding. Unraveling these regulatory intersections through integrative omics and gene editing tools will be instrumental in engineering unified resistance traits, enhancing crop resilience without compromising productivity.

## 4. Traditional Approaches to Disease and Pest Management in Peanut

For decades, peanut cultivation has relied on an array of conventional disease and pest management strategies rooted in the foundational principles of plant protection; avoidance, exclusion, eradication, protection, resistance, and treatment. These classical principles have guided generations of pathologists and agronomists in mitigating yield and quality losses caused by a diverse suite of pathogens and insect pests. An improved understanding of the disease triangle; host, pathogen, and environment has been central to refining these management frameworks and tailoring interventions for specific agroecological context [101].

### 4.1. Cultural Practices: The Cornerstone of Traditional Management

Among conventional strategies, cultural practices remain the most enduring and ecologically compatible tools for disease suppression in peanut systems. Crop rotation, early sowing, and soil tillage collectively disrupt pathogen life cycles and alter the soil microclimate, thereby curtailing the inoculum potential of soil-borne diseases [102]. Rotating peanuts with non-host crops such as cereals or bahiagrass has proven particularly effective in reducing the incidence of *Sclerotium rolfsii*, a major causal agent of stem rot, while simultaneously enhancing soil fertility and structure. Long-term field studies have demonstrated that bahiagrass-based rotations can improve peanut yield by up to 41% in comparison with continuous monocropping system [103], underscoring the synergistic benefits of ecological diversification and soil health restoration. Early planting is another crucial tactic, allowing crops to escape peak periods of pathogen or vector activity, while deep tillage reduces the survival of inoculum reservoirs in the rhizosphere. Collectively, these cultural interventions exemplify the preventive ethos of traditional plant protection managing disease pressure by manipulating the cropping environment rather than relying solely on reactive treatments.

### 4.2. Biological Control: Harnessing the Microbial Arsenal

Parallel to cultural methods, biological control has emerged as a sustainable avenue within traditional frameworks, leveraging naturally occurring antagonistic microorganism to suppress pathogens and pests [104]. Beneficial fungi such as *Trichoderma* spp., *Paecilomyces lilacinus*, and bacterial strains of *Bacillus* exhibit multifaceted biocontrol mechanisms, including mycoparasitism, competitive exclusion, antibiotic production, and induction of systemic resistance [105,106]. These microbes contribute not only to pathogen suppression but also to rhizosphere health, nutrient mobilization, and overall plant vigor.

The development and deployment of microbial biopesticides have further advanced the scope of biological control [107]. Agents such as *Bacillus thuringiensis* targeting lepidopteran pests and extracts from *Phytophthora* species after environmentally benign alternatives to synthetic pesticides [108]. Importantly, these biocontrol approaches align with the global shift toward eco-friendly agriculture by mitigating pesticide residues and preserving beneficial arthropod and microbial biodiversity.

### 4.3. Chemical Control: The Reactive Pillar of Conventional Management

Despite the growing emphasis on ecological solutions, chemical control remains a cornerstone of traditional peanut protection strategies especially when preventive measures fail or environmental conditions favour rapid disease proliferation. Broad-spectrum fungicides such as azoxystrobin, metam sodium, chlorothalonil are routinely used for immediate containment of fungal epidemics [109]. These compounds act rapidly to inhibit pathogen growth and prevent epidemic escalation, often serving as the last line of defense in integrated field management programs. However, the continued reliance on chemical inputs poses significant sustainability concerns. Intensive or improper fungicide application can accelerate the evolution of resistant pathogen populations, contaminate soil and water systems, and endanger non-target organisms, including beneficial microbes and pollinators. Moreover, the ecological and human health risks associated with chemical dependence are increasingly incompatible with modern sustainability goals.

## 5. Precision Breeding Tools for Peanut Disease and Insect Resistance

Conventional breeding has served as the foundation of peanut improvement, establishing the genetic foundation for resistance breeding programs over the past century (Table 3) [110]. Early breeders relied on systematic crossbreeding and phenotypic evaluations to incorporate resistance traits into cultivated varieties. These efforts were supported by extensive physiological research, field observations, and controlled experiments that clarified the inheritance and expression of resistance mechanisms. Through repeated cycles of hybridization and selection, elite peanut lines were developed with improved tolerance to major diseases and enhanced seed quality [111].

**Figure 2:**
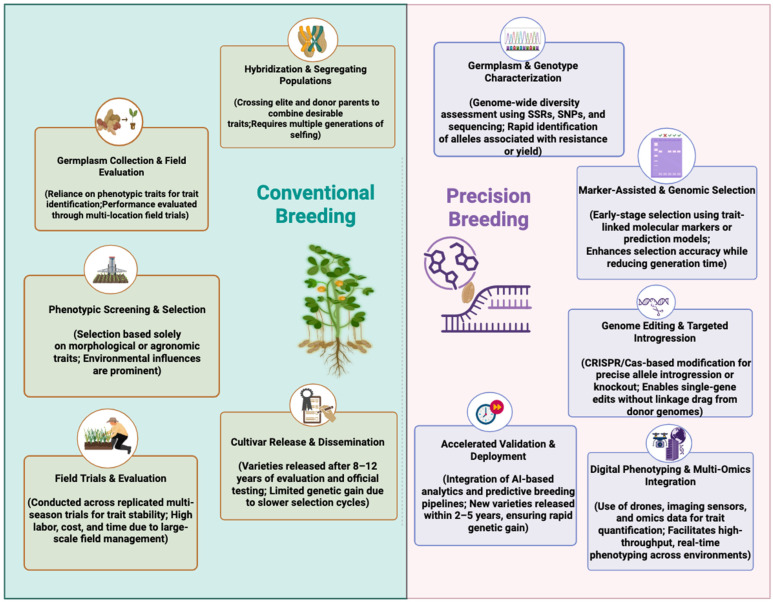
Comparative overview of conventional and precision breeding approaches in peanut (*Arachis hypogaea* L.) improvement. The left panel illustrates the traditional breeding pipeline, encompassing phenotypic selection, hybridization, and multi-season field evaluations, which are time-consuming and influenced by environmental variability. The right panel depicts the precision breeding framework integrating marker-assisted selection, genomic selection, genome editing, and digital phenotyping, enabling faster and more accurate development of pest- and disease-resistant cultivars. This visual comparison highlights how modern breeding technologies accelerate genetic gain and streamline resistance development in peanut.

Despite these accomplishments, the traditional breeding pipeline remains inherently time-demanding, typically extending over 7–12 years before a new cultivar reaches release (Figure 2). Its dependence on multi-season field trials and phenotypic selection, which are strongly influenced by environmental variation, slow genetic progress. Continuous breeding efforts are still essential to maintain desirable agronomic traits and integrate new value-added characteristics [112].

**Table 3:** Precision Breeding Tools and Their Applications in Peanut Improvement.

Technology/Tool	Application	Target Trait	Key Outcome	Reference
Marker-Assisted Selection (MAS)	Use trait-linked markers to introgressed or pyramid alleles (e.g., backcrossing high-oleic alleles or nematode/leaf-spot resistance QTLs into elite varieties)	High-oleic oil (AhFAD2), nematode resistance, major disease QTLs	Rapid introgression of single/few major loci with minimal linkage drag; proved route for high-oleic and specific resistance traits.	[113]
Genomic Selection (GS)	Genomic prediction models trained on marker panels to predict breeding values and select lines before phenotyping	Complex, polygenic traits such as quantitative disease tolerance, yield under stress	Improves selection accuracy and cycle time for complex traits; promising early results in peanut with ongoing methodological development.	[114]
Genome-Wide Association Studies (GWAS)/QTL Mapping	Scan diverse panels to find marker–trait associations and candidate genes for resistance	Late leaf spot, rust, other foliar diseases; putative NLR and PR proteins	Identifies loci and candidate genes (MQTLs) for downstream MAS, candidate gene validation, and genomic prediction.	[114]
CRISPR/Cas (Including Base Editors, Cas12a)	Targeted gene knock-out/knock-in or base editing (nutritional traits, virulence/host susceptibility genes, allergen reduction)	Candidate defense regulators (e.g., transcription factors), allergen genes, metabolic genes (AhFAD2 edits for oil quality)	Enables precise, targeted edits to validate gene function and create improved alleles (proof-of-concept demonstrated in peanut; hairy-root and tissue systems used).	[115]
RNA Interference (RNAI)/Host-Induced Gene Silencing (HIGS)	Silencing of pest or pathogen genes (or host susceptibility genes) using transgenic or topical RNA approaches	Insect pests (thrips, caterpillars), fungal virulence factors	Demonstrated reductions in pest/pathogen impact in experimental systems; useful for functional validation and targeted control strategies.	[116]
High-Throughput Phenomics (UAVS, Multispectral/Hyperspectral Sensors)	UAV/RGB/multispectral imaging and automated pipelines to measure canopy traits, NDVI, canopy temperature, LAI and stress signatures	Symptom severity for foliar diseases (late leaf spot), canopy vigor under pest/disease pressure, drought interactions	Rapid, repeatable field phenotyping that discriminates genotypes and supports downstream GWAS/GS/AI models.	[117]
Artificial Intelligence/Machine Learning (CNNS, RF, XAI)	Image processing, feature extraction, predictive models integrating phenomics, envirotyping and genomics for resistance prediction	Binary or probabilistic resistance classification; ranking breeding lines for advancement	Converts complex, multi-source data into actionable resistance scores and breeder decision support; improves selection efficiency when coupled with HTP.	[118]

Recognizing these limitations, researchers are now transforming peanut improvement through advanced molecular and genomic tools. Precision breeding approaches rooted in the identification of key resistance loci and the molecular dissection of complex traits enable faster, more accurate selection and trait introgression (Figure 2, Table 4). These innovations not only streamline breeding timelines but also enhance the efficiency and predictability of developing peanut cultivars with durable resistance to diseases and insect pests. Such next-generation strategies do not replace traditional practices but rather complement them embedding resilience within the genetic architecture of the crop while preserving ecological balance. The convergence of traditional wisdom and molecular innovations thus marks a paradigm shift from reactive management toward proactive resilience-building in sustainable peanut production systems. The subsequent discussion focuses in advanced molecular and genomic techniques driving progress in peanut breeding and genetics.

### 5.1. Marker-Assisted Selection for Polygenic Resistance Traits in Peanut

Majority of the agriculturally important traits in peanuts, including disease and insect resistance, exhibit a complex genetic architecture influenced by multiple genes and environmental interactions. Such traits, which display continuous variation across genotypes, are classified as quantitative traits [119]. Dissecting the genetic basis of these quantitative traits and precisely mapping the associated genomic regions are crucial steps toward targeted crop improvement. Molecular markers play a pivotal role in this process by serving as reliable proxies for specific loci inherited from parental lines. Marker-assisted selection (MAS) represents a powerful and efficient strategy for desirable alleles into elite germplasm, offering a faster and more precise alternative to traditional phenotypic selection [120,121]. The MAS pipeline can be further optimized by using seed-derived DNA instead of leaf tissue, allowing partial seed sampling for genotyping while preserving the reminder for germination. Over the years, a variety of molecular markers such as restriction fragment length polymorphism (RFLP), amplified fragment length polymorphism (AFLP), random amplified polymorphic DNA (RAPD), cleaved amplified polymorphic sequence (CAPS), and simple sequence repeat (SSR) markers have been employed in peanut breeding programs [122].

Among these, SSR markers stand out for their co-dominant inheritance, high reproducibility, locus specificity, and broad genomic distribution. Consequently, they have become the preferred tool for mapping and introgressing disease resistance QTLs across multiple crops, including peanut [123,124]. Extensive use of SSR-based linkage mapping has enabled the identification of major QTLs conferring resistance to several key peanut pathogens. In two recombinant inbred line (RIL) populations derived from Tifrunner × GT-C20 (T population) and SunOleic 97R × NC94022 (S population), two major QTLs governing tomato spotted wilt virus (TSWV) resistance were mapped [122]. Later, the construction of an improved SSR-based genetic map from the T population led to the identification of six QTLs for early leaf spots (ELS), five for late leaf spot (LLS), and one for TSWV resistance [123].

Fine mapping with additional SSR markers furthers refined a major QTL for TSWV resistance, narrowing the genomic interval and explaining 22.8% of the phenotypic variance [125]. Similarly, analysis of F_2_ lines derived from the cross GG 20 (susceptible cultivar) × CS 19 (resistant interspecific pre-breeding line) uncovered a stem rot resistance QTL, *qstga01.1*, accounting for over 17% of the phenotypic variation. SSR markers have also facilitated the mapping of QTLs associated with bacterial wilt rust, and leaf spot resistance. Specific SSR alleles identified in crosses ICGV99005 × TMV2 and ICGV99003 × TMV2 were found to be linked with rust resistance [126]. In another study, RIL population derived from *VG 9514* (rust-resistant) × TAG 24 (susceptible) was evaluated under field conditions, resulting in the construction of a genetic linkage map containing 24 linkage groups with 109 SSR markers. Particularly, rust resistance was mapped to linkage group 2, where the SSR marker gi56931710 was positioned at 3.4 cM, while pPGPseq4A05 was located at and 4.7 cM [127].

Recent advancements continue to expand the scope of MAS in peanut improvement. For instance, identified additional QTLs influencing aflatoxin accumulation: qAFTB05.2, qAFTA05.1, and qAFTB06.3 and revealed additive genetic effects through conditional mapping. Complementing this, Yu et al. [128] reported a consistent QTL, qAFTsA07.1, accounting for 13.39% of phenotypic variation. These findings underscore the growing precision and applicability of MAS in dissecting complex resistance traits, paving the way for accelerated genetic gains and the development of resilient peanut cultivars.

### 5.2. Genome-Wide Association Study (GWAS) for Dissecting Resistance Traits in Peanut

Genome-wide association study (GWAS) have transformed the landscape of crop genetics by enabling the dissection of complex, polygenic traits at a much finer resolution than traditional linkage mapping. Instead of relying on controlled crosses, GWAS leverages natural variation within diverse germplasm collections and exploits linkage disequilibrium (LD) the non-random correlation between alleles at different loci to pinpoints genomic regions associated with trait variation [129,130]. This approach has proven particularly powerful for identifying allelic variants and candidate genes linked to disease resistance, thereby supporting marker-assisted and genomic breeding programs aimed at developing resilient peanut varieties optimized for distinct agroecological zones [131].

By integrating historical recombination events accumulated over many generations, GWAS achieves superior mapping resolution and can detect loci influencing traits with low penetrance or subtle phenotypic effects. In peanut, the first large-scale GWAS conducted on 120 genotypes mainly from the US peanut mini-core collection successfully identified two major QTLs conferring resistance to both LLS and ELS, along with two additional ELS-specific QTLs [114]. Expanding this approach, Achola et al. [132] reported 32 marker-trait associations (MTAs) and revealed a potential *TIR-NBS-LRR* gene on chromosome A04 linked with broad-spectrum disease resistance. Similarly, GWAS targeting stem rot resistance uncovered 27 candidate defense related genes implicated in pathogen recognition, signalling cascades and immune response activation. Nevertheless, the practical deployment of GWAS in peanut improvement faces several constraints. Large-scale analyses require extensive genotypic and phenotypic datasets encompassing high genetic diversity, which are not always available in recently developed breeding programs. The peanut genome being an allotetraploid further complicates association analyses by blurring the distinction between homologous and homoeologous loci. Moreover, GWAS signals often localize to intergenic or non-coding regions, which complicates the interpretation of functional relevance [133]. The high cost of whole-genome sequencing also limits its widespread use, and while SNP arrays provide a more affordable alternatives, predesigned panels may overlook novel or rare variants critical for adaptive resistance. Despite these limitations, GWAS remains indispensable for uncovering the genetic basis of quantitative resistance in peanut. Integrating GWAS with multi-omics platforms such as transcriptomics and metabolomics, alongside improved genome assemblies, could significantly accelerate the identification of functional alleles and enhance precision breeding pipelines for durable disease and pest resistance.

### 5.3. High-Throughput Phenotyping in Peanut

The advent of high-throughput phenotyping (HPT) has transformed the way complex traits are evaluated in peanut breeding programs, particularly those related to disease and pest resistance. Many major pathogens and insect pests cause extensive damage by reducing the total leaf or photosynthetic area, making precise quantification of canopy traits essential for assessing plant performance. However, traditional manual methods for estimating such parameters have long been labor-intensive and prone to measurement inconsistencies. Before the emergence of phenomics-based approaches demonstrated early efforts to estimate peanut canopy leaf area by correlating leaf and total mass measurements across multiple cultivars and growth stages. The transition to automated phenotyping has since revolutionized this process. With the integration of imaging technologies, 3D reconstruction, and automated data pipelines, researchers can now evaluate large breeding populations with remarkable speed and precision [134,135].

Field-based applications of HTP have also gained attention in peanut improvement programs. UAV-mounted sensors to capture vegetative indices and canopy temperature across a chromosome segment substitution line (CSSL) population, successfully identifying lines exhibiting resistance to ELS, and LLS. Notably, CSSL lines 100, 84,111, and 15 showed a significantly lower incidence of TSWV. The study further integrated ground penetrating radar (GPR) to estimate pod yield non-destructively prior to harvest, illustrating how multi-sensor systems can enhance selection accuracy in field breeding trials.

Complementary advances in predictive analytics have also strengthened phenotyping capacity. Sarkar et al. [117] employed aerial imaging combined with multiple linear regression and artificial neural network (ANN) models to estimated peanut LAI and lateral growth with accuracies ranging from 84 to 97 per cent. Such models exemplify how data-driven approaches can bridge the gap between physiological traits and genetic improvement. Looking ahead, HTP is poised to transform peanut breeding by enabling rapid, non-invasive, and scalable assessment of plant-pathogen interactions. Its integration into breeding pipelines could significantly accelerate the discovery of resistant genotypes and the development of climate-resilient, high-yielding cultivars, thereby strengthening global food security amid rising biotic stress challenges.

**Table 4:** Key Markers and Gene-Edited Targets Applied in Practical Breeding.

Category	Crop/Species	Gene/Marker	Trait Improved	Reference
MAS	*Oryza sativa*	Pi9 + Pi54 functional markers	Blast disease resistance	[136]
MAS	*Oryza sativa*	Gn1a and other major QTLs	Grain number and yield	[137]
MAS	*Triticum aestivum*	TaGW2, TaSus1	Grain weight/quality	[138,139]
GENOME EDITING	*Zea mays*	ARGOS8 (CRISPR-generated variant)	Enhanced yield under stress	[140]
GENOME EDITING	*Oryza sativa*	OsERF922 (CRISPR/Cas9)	Resistance to *Magnaporthe oryzae* (Rice blast)	[141]
GENOME EDITING	*Oryza sativa*	IPA1, GS3, DEP1, Gn1a (CRISPR/Cas9)	Yield, panicle architecture	[142]
GENOME EDITING	*Glycine max*	FAD2-1A and FAD2-1B (CRISPR/Cas9)	Increased oleic acid (oil quality)	[143]
GENOME EDITING	*Solanum lycopersicum*	SlIAA9 (CRISPR/Cas9)	Fruit quality/seed lessness	[144]
Genome Editing	*Cucumis sativus*	eIF4E (CRISPR/Cas9)	Resistance to Cucumber vein yellowing virus (Ipomovirus)	[145]
Genome Editing	*Glycine max*	*GmPDCT* (CRISPR/Cas9)	Oil quality (fatty acid profile)	[146]
Genome Editing	*Glycine max*	*GmTAP1* (CRISPR/Cas9)	Root rot resistance	[147]
Genome Editing	*Solanum lycopersicum*	*SlMLO1* (CRISPR/Cas9)	Powdery mildew resistance caused by *Oidium neolycopersici*	[148]
Genome Editing	*Hordeum vulgare*	Barley MLO (CRISPR/Cas9)	Powdery mildew resistance	[149]
Genome Editing	*Solanum lycopersicum*	Pmr4 (CRISPR/Cas9)	Powdery mildew resistance	[150]
Genome Editing	*Citrus sinensis*	CsLOB1 (CRISPR/Cas9)	Resistance to citrus canker	[151,152]
Genome Editing	*Musa* spp.	RGA2, Ced9 (CRISPR/Cas9)	Resistance to Fusarium wilt	[153]
Genome Editing	*Grape vine*	VvWRKY52 (CRISPR/Cas9)	Resistance to B. cinerea	[154]
Genome Editing	*Solanum tuberosum* L.	StSR4	Resistance to P. infestans	[155]
Genome Editing	*Capsicum annuum*	CaERF28	Resistance to Anthracnose disease	[156]
Genome Editing	*S. tuberosum* L.	Nib, CI, CP, and P3 conserved viral regions (CRISPR/Cas13a)	Resistance to Potato virus Y	[157]
Genome Editing	*Brassica napus*	BnCRT1a	Resistance to Verticillium longisporum (Vl43)	[158]

## 6. Advances in Disease and Insect Resistance Breeding

### 6.1. Genetic Engineering Strategies for Peanut Disease and Insect Resistance

Genetic engineering has emerged as a powerful approach to enhance disease and insect resistance in peanut, complementing conventional breeding through the precise integration of defense-related genes into elite cultivars. Core transformation systems, including *Agrobacterium*-mediated gene transfer and biolistic delivery, have facilitated stable transgene expression and functional trait improvement. The foundation of peanut genetic transformation was established in the early 1990s with the pioneering works of Ozias-Akins, who demonstrated transformation via particle bombardment who successfully introduced the peanut strip virus coat protein gene through *Agrobacterium*-mediated transformation. These breakthroughs catalysed the development of genetically engineered peanuts with enhanced resistance to major pathogens and insect pests. This progress by engineering pest-resistant and aflatoxin-reducing transgenic peanuts, highlighting the potential of *Bacillus thuringiensis* (*Bt*)-derived *Cry* genes. These genes encode insecticidal crystal proteins that provide protection against a wide range of lepidopteran pests. The introduction of *CryIA*(c) conferred resistance to the lesser cornstalk borer, while *Cry1AcF*, and *Cry1EC* imparted strong defense against *Spodoptera litura* [159]. Dual gene constructs, such as *cry1EC + Chi11*, provided combined resistance to *S. litura* and *Phaeoisariopsis personata*, and *cry1X* effectively targeted defoliators *Spodoptera litura* and *Helicoverpa armigera*. Similarly, *cry8Ea1* and *cry8Ea1* + *MARs* transgenic lines displayed improved resistance to the *Holotrichia parallela* (white grub) [160].

Fungal resistance has also been significantly strengthened through the incorporation of chitinase and antifungal protein genes. Transgenic lines expressing chitinase gene from tobacco, and rice exhibited resistance against early and late leaf spot diseases caused by *Cercospora arachidicola* and *P. personata* [94]. Expression of *SniOLP* (from *Solanum nigrum*) and Rs-AFP2 (from *Raphanus sativus*) further enhance fungal resistance. Similarly, β 1–3 glucanase from *Arabidopsis*, alfalfa, and tobacco have shown broad-spectrum antifungal efficacy [161], while *AdSGT1* from *Arachis diogoi* strengthened defense signalling against *P. personata*. Moreover, genes conferring oxidative stress tolerance have been exploited to counter soil-borne fungal pathogens. The expression of barley oxalate oxidase improved resistance to *Sclerotinia* minor, the causal agent of *Sclerotinia* blight [162]. Together, these genetic engineering interventions underscore the success of transgenic technologies in peanuts for building durable, multi-pathway resistance to both diseases and insect pests, laying the groundwork for future precision breeding and genome editing innovations.

### 6.2. CRISPR/Cas9-Mediated Gene Editing for Disease and Insect Resistance

The effectiveness of genome editing in crop plants usually hinges on the strength of the underlying transformation and regeneration framework, which itself is shaped by a complex interplay of biological and technical variables, including genetic background, explant origin, *Agrobacterium* strain compatibility, selection pressure, culture media formulation, environmental conditions, and hormonal regulation [163]. In peanut, a tetraploid legume widely recognized for its transformation recalcitrance, these constraints are particularly pronounced making reliable plant regeneration a persistent obstacle. Over the years, extensive efforts have been devoted to identifying responsive target tissues, leading to the evaluation of a diverse array of explants, ranging from leaflets, hypocotyls, epicotyls, and axillary buds to mature and immature cotyledons, cotyledonary nodes, embryonic axes, in-planta seedling systems, and embryogenic calli [164,165,166,167,168,169,170]. Among the tested systems, de-embryonated cotyledons have emerged as one of the most responsive explant types, repeatedly delivering comparatively high transformation efficiencies, in some cases approaching 58–81% [171,172]. Nevertheless, the overall low transformation efficiency, instability of transgenic events, and labour-and time-intensive nature of existing protocols have substantially constrained progress in peanut gene functional analysis and trait engineering.

Against this backdrop, the advent of CRISPR/Cas9-baed genome editing has revolutionized plant biotechnology by enabling precise, efficient, and targeted modification of specific genes to develop desirable agronomic traits. Unlike conventional breeding or transgenic approaches, CRISPR/Cas9 allows for the direct manipulation of native genomes, offering unparalleled control over trait expression and rapid generation of improved crop varieties [173]. In crop improvement programs, CRISPR/Cas9-induced loss-of-function mutations have proven particularly useful for uncovering gene function and converting susceptible alleles into resistant ones. Recent advances in CRISPR/Cas9-mediated genome editing have led to successful genetic improvement across a wide range of crop species including barley (90), *Arabidopsis* (89), maize (92), *Brassica napus* (91), rice (93), peanut (6), sorghum (94), wheat (98, 99), and soybean (95–97).

For peanut improvement, CRISPR/Cas9 holds immense promise in targeting genes underlying susceptibility to fungal and insect pests, traits that have been difficult to address through conventional breeding alone [128]. Moreover, genome editing extends beyond plant systems; recent studies suggest its potential for regulating insect pest populations through genetic manipulation, thereby reducing crop losses and dependence on chemical pesticides [174]. Altogether, CRISPR/Cas9-based editing represents a transformative platform in precision breeding, offering a sustainable and scalable solution to enhance peanut resilience against biotic stresses while maintaining yield and quality.

#### 6.2.1. CRISPR/Cas9 Applications in Plant Disease Resistance and Pathogen Detection

The emergence of CRISPR/Cas9 technology has revolutionized modern plant biotechnology, offering an unprecedented level of precision in editing genes that regulate host-pathogen interactions. This system allows targeted modification of susceptibility (S) genes and fine tuning of defense signalling networks, ultimately enhancing plant immunity and shaping the future of sustainable crop protection [175]. Its versatility extends beyond plants to include editing of pathogen genomes themselves, opening new frontiers for developing both resistant cultivars and weakened pathogen strains for research and management purposes. The CRISPR/Cas platform has been successfully deployed across a wide range of plant systems from model organisms to cereals and horticultural crops demonstrating its adaptability and efficiency in generating heritable gene edits [176]. Moreover, its application in fungi and oomycetes has led to fundamental discoveries in pathogenicity and virulence mechanisms, with genome edits achieved in *Aspergillus* spp., *Alternaria alternata*, *Ashbya gossypii*, *Ustilaginoidea virens*, *Ustilago maydis*, *Fusarium fujikuroi*, *F. graminearum*, *F. oxysporum*, *M. oryzae*, *Leptosphaeria maculans*, *Phytophthora capsica*, *P. sojae*, *P. palmivora* [176], *Sclerotinia sclerotiorum*, *Shiraia bambusicola*, *Ustilago tricophor*, and *Huntiella omanensis*, among others. These advances highlight the technology’s potential to dissect pathogen biology and uncover novel targets for crop resistance engineering [177].

In crop improvement, CRISPR/Cas-mediated editing of susceptibility genes has emerged as a keystone strategy for engineering durable resistance [178]. A classic example involves disruption of the MLO gene which was originally discovered in barley whose orthologs in wheat and tomato, when edited, conferred strong resistance to powdery mildew infection [148]. Similarly, in wheat, simultaneous knockout of three *TaEDR1* homologs through CRISPR/Cas9 enhanced resistance to the same pathogens by modulating defense signalling pathways. Resistance against viral pathogens has also been achieved via editing of the eIF4E gene family, a key susceptibility determinant for potyvirus infections in cassava, *Arabidopsis*, and cucumber [179,180]. Furthermore, targeted mutagenesis of *TaNFLX1* in wheat limited *F. graminearum* infection and mycotoxin accumulation, exemplifying how CRISPR-mediated mutagenesis can mitigate fungal diseases [181].

Beyond its genome editing role, CRISPR/Cas systems have transformed pathogen diagnostics, offering ultra-sensitive and specific detection of DNA and RNA molecules. Cas nuclease-based assays, such as those employing Cas12a and Cas13, enable rapid, field-deployable detection of plant pathogens, significantly improving disease surveillance and management [182]. Cas12a-based detection platforms have been applied for identifying *Magnaporthe oryzae*, the causal agent of rice blast [183], and for visual detection of *Grapevine red-blotch* virus, apple RNA viruses, and viroids. While CRISPR/Cas-based applications in peanut pathology remain limited, the progress achieved in other crop systems provides a strong foundation for future exploration. Integrating CRIPSR-driven resistance engineering with precision diagnostics hold the potential to transform peanut disease management, offering rapid detection, targeted intervention, and sustainable resistance solutions against evolving pathogens.

#### 6.2.2. CRISPR/Cas9 Strategies for Insect Resistance Management

Endogenous resistance to insect pest in peanut is mediated by molecular defense pathways that are particularly critical for underground herbivores, where resistance is predominantly quantitative, root-localized, and tightly regulated at the transcriptional level. Evidence from wild *Arachis* species demonstrates that resistance to soil-dwelling pests, including root-knot nematodes, is associated with the activation of immune receptor genes such as *NBS-LRR* family members and the induction of jasmonic acid (JA)-dependent signalling cascades, resulting in cell wall reinforcement and enhanced accumulation of defensive secondary metabolites in root tissues [184,185]. Comparative transcriptomic analyses further reveal that key transcription factors, notably *WRKY* and *MYB*, function as regulatory hubs controlling phenylpropanoid and flavonoid biosynthesis pathways that directly impair herbivore feeding and development, a conserved defense strategy across legume and other plant species [186,187]. Despite their effectiveness, the translation of these endogenous mechanisms into breeding gains remains limited by their polygenic architectures, strong genotype × environment interactions, and the technical challenges of accurately phenotyping subterranean insect damage under field conditions.

In this context, CRISPR/Cas genome editing offers a transformative alternative to conventional breeding and transgenic approaches by enabling precise genetic modifications that reprogram plant-insect interactions at the molecular level. By modifying effector-target recognition, silencing host-susceptible genes, or decoupling the antagonistic crosstalk between growth and defense signalling pathways, genome editing opens novel avenues for developing crops with enhanced insect resistance [188].When natural resistance (R) genes are limited or absent, CRISPR/Cas technology provides the possibility of creating de novo resistance by directly introducing targeted mutations into susceptibility loci [189]. Gene editing has also enabled novel approaches to insect population control, such as the induction of sterility, disruption of reproductive genes, and deployment of gene drives designed to spread deleterious alleles throughout pest populations. Such interventions are particularly valuable as resistance to *Bacillus thuringiensis* (*Bt*)-derived toxins continues to emerge, challenging the long-term efficacy of *Bt* crops. Consequently, CRISPR-Cas9 has become a leading technology for designing next-generation insect management strategies that are both economically feasible and ecologically responsible [190].

Functionally, CRISPR-based genome editing modifies specific DNA sequences by introducing, deleting, or replacing nucleotides to disrupt gene function or create novel traits. Recent insect studies have demonstrated its precision and utility. For example, CRISPR/Cas9-mediated knockout of the *NPC1b* gene in *Helicoverpa armigera* larvae revealed its essential role in dietary cholesterol uptake and growth, where cholesterol assimilation led to stunted development and diminished feeding activity. This highlights *NPC1b* as a promising target for insect control, though off-target effects remain a concern. Similarly, gene disruption of *HaCad*, a receptor for *Cry1Ac* toxin, using CRISPR/Cas9 established its role in *Bt* resistance, suggesting pathways to counteract resistance development in field populations [191].

Furthermore, CRISPR-based manipulation of reproductive and developmental genes has yielded new biological control strategies. In *H. armigera*, targeted modifications of mating-related genes reduced fecundity and altered mating rhythms, offering a non-chemical pest suppression mechanism. In *Spodoptera litura*, disruption of *Slabd-A* caused severe defects in segmentation and pigmentation during embryogenesis, while knockout of *litBLOS2* eliminated larval pigmentation patterns such as yellow stripes and white spots, providing visible markers for functional genomic studies. The earliest successful demonstration of CRISPR/Cas9 in a storage pest, *Tribolium castaneum*, underscored the broad applicability of this tool across insect taxa [192]. These studies collectively exemplify the growing promise of CRISPR/Cas-mediated genome editing as a frontier technology for insect management. By integrating precise genetic interventions in both crops and pest genomes, this approach can substantially reduce reliance on chemical insecticides and usher in a new era of eco-friendly pest control strategies.

#### 6.2.3. CRISPR/Cas-Mediated Crop-Level Innovations for Insect Resistance

CRISPR-Cas technologies have opened a transformative avenue for enhancing plant vigor and fortifying crops against diverse biotic and abiotic challenges. Beyond the direct modulation of pest resistance genes, gene editing can also influence ecological interactions, for instance, modifying volatile compounds to repel insect pests or attract natural enemies that suppress their populations. A classic demonstration by Beale et al., [193] revealed that engineering volatile emissions such as the *sesquiterpene hydrocarbon E-β-farnesene* (*Eβf*) could simultaneously reduce aphid feeding and recruit parasitic wasps (*Diaeretiella rapae*), thus creating a self-sustaining biological control mechanism. These findings highlight how manipulating volatile blends via CRISPR-based tools could establish eco-friendly pest control strategies, though caution remains essential to avoid adverse impacts on beneficial insect communities.

At the molecular level, genome editing enables precise modulation of plant immunity components particularly the balance between resistance (R) genes and susceptibility (S) genes to achieve durable insect tolerance. R genes govern host recognition and resistance, whereas S genes render plants vulnerable to pests and pathogens; thus, targeted modifications of these loci through CRISPR offers a rational path to enhanced defense. This concept has been effectively demonstrated in rice, where disruption of the *CYP71A1* gene via CRISPR-Cas9 reduced *tryptamine 5-hydroxylase.* activity, shifting tryptamine metabolism toward serotonin accumulations and significantly hindering the growth of plant hoppers. Similarly, genome editing in *Populus tomentosa* achieved simultaneous knockouts of *PtoPDS1* and *PtoPDS2*, confirming the feasibility of CRISPR-based modification for pest management in woody perennials [194].

Plant visual traits also play a pivotal role in insect host selection. Altering pigmentations pathways has proven to disrupt insect feeding behaviour and preference. In red-leaf tobacco, the manipulation of anthocyanin biosynthesis altered leaf coloration, reducing recognition by *Helicoverpa armigera* and *Spodoptera litura*. This illustrates how CRISPR-mediated engineering of pigment pathways can indirectly confer pest tolerance by interfering with host detection mechanisms. Similar progress has been made in soybean, where CRISPR-induced deletions in *GmCDPK38* (*Hap3*) increased resistance to common cutworms, and mutations in the *GmUGT* gene conferred tolerance to *S. litura* and *H. armigera* [114]. More recently, multiplex CRISPR-Cas9 editing of 2S albumin allergen genes in peanut achieved targeted protein elimination, marking an initial step toward broader functional and resistance-oriented genome modification [195]. However, the application of CRISPR/Cas9 for insect resistance improvement in peanut remains in its infancy, primarily constrained by transformation efficiency and limited genomic resources.

A major technological bottleneck lies in the efficient delivery of CRISPR/Cas reagents and subsequent plant regeneration. Conventional tissue culture-based systems are time-consuming and genotype-dependent. Cutting edge alternatives, including nanomaterial-assisted delivery and viral vector-mediated in planta genome editing, are emerging as promising routes to overcome these challenges [196]. These developments underscore the next frontier of CRISPR/Cas applications wherein crop-level genome editing transcends laboratory confines to become an integral part of sustainable pest management in agricultural ecosystems.

#### 6.2.4. Viral Vector-Mediated Delivery of CRISPR Components in Plants

To further enhance the efficiency and applicability of CRISPR-based genome editing, particularly in recalcitrant crops, plant-virus-based vectors have emerged as powerful delivery platforms [197,198]. Because of their limited cargo capacity, viral vectors are most commonly used to deliver CRISPR single-guide RNA (sgRNA) that act in plants constitutively expressing Cas9, although split or minimized nuclease systems have also been reported [199,200,201]. Importantly, positive strand RNA viruses such as tobacco rattle virus (TRV) and barley stripe mosaic virus (BSMV), can move systematically into meristematic tissues, enabling heritable genome edits without tissue culture [196,202,203,204]. These systems have been successfully applied to edit genes associated with pathogen susceptibility and defense regulation, providing effective control of plant diseases, and offer a conceptual framework for targeting genes involved in insect-plant interactions to reduce pest damage [205]. Although viral vectors are attractive tools for genome editing in plant because of their highly efficient and systemic infection; however, their use requires careful vector engineering to accommodate additional DNA or RNA cargo [206]. Most viral vectors are derived from insect-transmitted plant viruses, and the host specificity of these insect vector inherently restricts delivery to a narrow range of plant species. Moreover, some commonly used host plants accumulate secondary metabolites that interfere with the purification of recombinant products, limiting their suitability for downstream applications [206]. Consequently, the development of viral vector systems in metabolically favourable host plants with low levels of undesirable compounds represents a promising avenue for enhancing product purity and commercial acceptance.

#### 6.2.5. Nanotechnology-Assisted Delivery of Genome Editing in Biotic Stresses

Nanotechnology has become an integral component of modern biological, medicinal, and pharmaceutical research and is increasingly influential in plant science, particularly in the context of genome editing for crop improvement [207,208,209]. By enhancing resilience to biotic and abiotic stresses and improving crop yield and quality, nanotechnology aligns closely with the objectives of CRISPR/Cas9-based precision breeding [210,211]. Nanoparticles function as efficient carriers for the delivery of genetic materials into plant and animal cells and specific tissues, with established application in the development of desirable agronomic traits in crops [212]. The mechanistic basis and practical implementation of nanoparticle-mediated gene transformation have been well-described, highlighting both the advantages and limitations of these systems compared with conventional delivery methods [213,214]. A wide spectrum of nanoparticles platforms including mesoporous silica nanoparticles, carbon nanotubes, gold nanoparticles, and magnetic nanoparticles has been successfully exploited to deliver plasmid DNA, double-stranded RNA, and small interfering RNA (siRNA) into plant protoplasts and intact tissues using diverse delivery strategies [212,215,216,217,218]. Remarkably, magnetic nanoparticle mediated pollen transformation has enabled effective gene silencing and genome editing in plants, providing a tissue-culture-independent route for genetic manipulation [213]. Complementary approaches using small nanoparticle-CRISPR/Cas9 vector complexes microinjected into leaves or other plant organs have further demonstrated the feasibility of edited plants that can be readily propagated through tissue culture or simplified regeneration protocols [213,219]. In parallel, carbon dot-siRNA complexes have been shown to silence reporter genes such as GFP in tobacco and tomato, underscoring the versatility of nanocarriers for RNA-based regulation in plants [220]. More recently, the integration of nanoparticle delivery systems with CRISPR/Cas9 has enabled stable genome editing accompanied by clear regeneration and phenotypic or metabolic alterations, reinforcing the potential of nanotechnology-driven genome engineering as a scalable tool for crop improvement and for targeting traits relevant to biotic stresses [211].

## 7. AI-Driven Remote Sensing for Smart Disease Surveillance in Peanut

The integration of remote sensing and artificial intelligence (AI) has revolutionized precision agriculture, offering scalable, non-destructive, and real-time tools for crop health monitoring. Remote sensing encompasses the acquisition, visualization, and analysis of image-based data that reflect the physiological and pathological status of crops across various growth stages. In peanuts, these technologies are increasingly leveraged for accurate, cost-effective, and rapid disease diagnosis [221], representing a major step toward data-driven disease management systems.

Ground-based sensing platforms, equipped with RGB, hyperspectral, multispectral, and thermal cameras, have proven invaluable for diagnosing peanut foliar diseases such as early and late leaf spots, southern blight, stem rot, and leaf wilting. These optical sensors capture disease-specific spectral variations, allowing early detection before visible symptoms fully manifest. RGB imagery enables quantification of disease-induced changes such as chlorosis, necrosis, and lesion expansion, while computer vision and machine learning algorithms process these images to discriminate between healthy and diseased tissues with high accuracy. Moreover, high throughput phenotyping (HTP) systems that employ RGB-based imaging pipelines have accelerated data collection and analysis in peanut breeding programs, facilitating large-scale screening for disease tolerance [222].

HTP has emerged as a cornerstone of AI-driven crop improvement, enabling raid, accurate, and large-scale assessment of plant traits related to growth, yield, stress tolerance, and disease resistance [223,224]. Unlike traditional manual phenotyping, which is labour-intensive and error-prone, HTP integrates advanced imaging, remote sensing, robotics, and sensor technologies to generate objective and repeatable phenotypic data across temporal and spatial scales [225,226]. Advances in sensor technology, robotics and autonomous platforms such as unmanned aerial vehicles (UAV), ground-based vehicles, and agricultural robots now allow simultaneous, non-destructive measurement of multiple traits across large breeding populations within short timeframes [227,228,229,230,231]. Integrating UAV- and sensor-derived phenomic data with genomic information has been shown to substantially enhance the performance of genomic selection models for complex traits. In cereals and tuber crops, the incorporation of vegetation indices, spectral reflectance, and thermal traits alongside genome-wide markers using multitrait and multikernel prediction frameworks has consistently improved prediction accuracy and robustness across environments [232,233,234,235]. Similar AI-enabled phenomic-genomic integration holds strong potential for improving selection efficiency for quantitative disease and pest resistance in peanut, enabling earlier identification of resilient genotypes and accelerating genetic gain in modern breeding programs.

Hyperspectral imaging, with its broader wavelength sensitivity, further refines peanut disease scouting and forecasting. By analyzing canopy reflectance, particularly in the near-infrared (NIR) range (750–950 nm), hyperspectral sensors can detect subtle physiological changes associated with disease onset, including reduced chlorophyll and altered water content [236]. Field-based instruments such as the ASD Field Spec3 spectrometer have achieved up to 96.88% accuracy in detecting peanut leaf spot symptoms (at 570, 671, and 750 nm) and 90.5% accuracy in assessing southern blight severity. Similarly, the Jaz spectrometer, when integrated with machine learning algorithms, has demonstrated over 90% accuracy in stem rot detection using optimal wavelengths ranging from 501–884 nm. These findings affirm that hyperspectral imaging, combined with AI analytics, is a reliable approach for early and precise peanut disease surveillance. Despite their high accuracy, ground-based sensing systems are limited in coverage and scalability, particularly over large or uneven agricultural fields. UAVs equipped with multispectral or hyperspectral cameras, have emerged as an advanced alternative capable of high-resolution spatial and temporal monitoring [237]. UAV-based imaging enables the rapid assessment of peanut field health and the detection of diseases such as bacterial wilt through variations in leaf reflectance signatures where reduced water and chlorophyll content correspond with declining NIR reflectance values. Beyond monitoring, UAV integration with precision spraying systems facilitates site-specific agrochemical applications, ensuring accurate treatment of diseased zones and minimizing chemical overuse [236].

Looking ahead, the convergence of AI-driven analytics, advanced spectral sensors, and UAV-based delivery systems represents the next frontier in peanut disease management. AI algorithms, including deep learning and convolutional neural networks, can further enhance pattern recognition and predictive modelling of disease outbreaks, thereby supporting early intervention strategies. The development of autonomous and variable-rate smart sprayers capable of interpreting sensor feedback in real time will enable targeted disease suppression with exceptional precision [103]. To fully realize the potential of these innovations, future research must focus in creating integrated frameworks that combine ground-based sensing, UAV surveillance, and AI-based decision-support systems. Such holistic, data-fused platforms could significantly reduce disease-induced yield losses, promote sustainable peanuts production, and contribute to the broader goal of intelligent, climate-resilient agriculture.

## 8. Multi-Omics Integration for Deciphering Disease Resistance Mechanisms in Peanut

The integration of multi-omics technologies is revolutionizing the understanding and improvement of disease resistance in peanut by bridging the gap between genotype and phenotype. While genomic selection (GS) has greatly accelerated breeding progress through the use of genome-wide DNA markers, particularly single-nucleotide polymorphisms (SNPs), to predict genomic estimated breeding values (GEBVs), its predictive power remains limited for polygenic traits governed by complex molecular interactions. Disease resistance, being a highly dynamic trait, involves intricate regulatory networks encompassing transcriptional, translational, and metabolic responses to pathogen attack [238]. Consequently, relying solely on genomic data cannot fully capture the multi-layered biological responses that determine the plant’s defense capacity.

To overcome this limitation, researchers are increasingly adopting multi-omics frameworks that integrate genomics, transcriptomics, proteomics, metabolomics, and epigenomics to construct a holistic view of trait regulation. By linking molecular signatures from multiple biological layers, these approaches enable more precise identification of regulatory genes, signalling pathways, and metabolic shifts associated with disease resistance [239]. The combination of omics data enhances the resolution of genotype-phenotype associations, facilitating the discovery of biomarkers and predictive models that capture both static genetic variation and dynamic stress-responsive processes.

**Figure 3:**
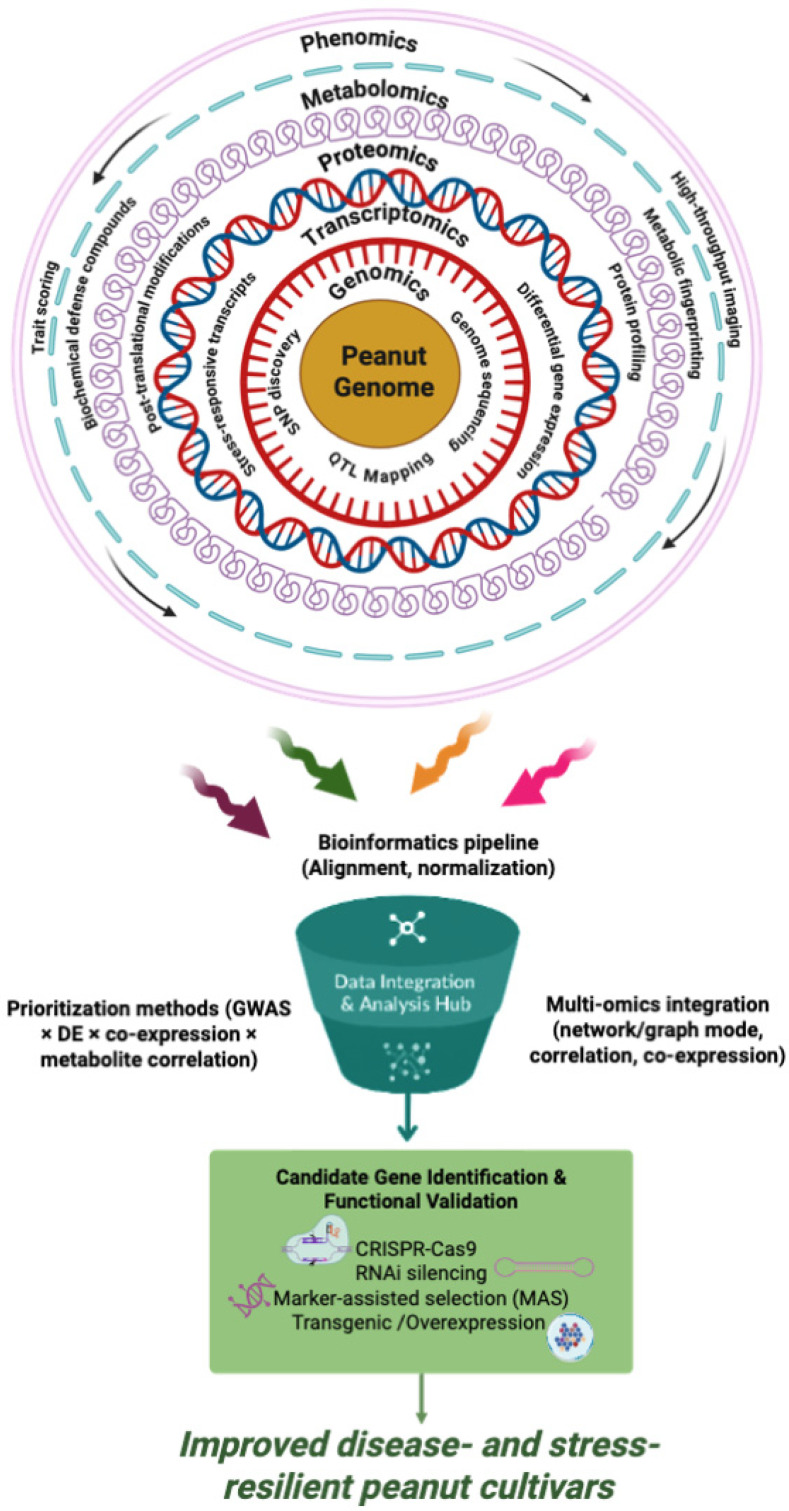
Integration of multi-omics platforms for candidate gene discovery and crop improvement in peanut (*Arachis hypogaea* L.). This schematic illustration represents the convergence of genomics, transcriptomics, proteomics, metabolomics, and phenomics datasets to uncover key candidate genes associated with complex agronomic traits in peanut. Through data integration and computational analysis, gene candidates are prioritized for functional validation using tools such as CRISPR/Cas9-mediated editing, RNA interference (RNAi), and marker-assisted selection (MAS). The integration of these approaches accelerates the development of stress-resilient, high-yielding peanut cultivars, thereby supporting modern precision breeding programs.

Multi-omics integration provides a systems-level understanding of plant defense beyond single-marker associations (Figure 3). Genomic datasets reveal structural and sequence variations conferring resistance; transcriptomics elucidates gene expression dynamics under infection; proteomics uncovers post-translational modifications critical for signalling; metabolomics highlights the role of defense metabolites; and phenomics link these molecular insights to observable resistance phenotypes [239]. Together, these data layers provide a comprehensive map of defense architecture, revealing how peanut plants orchestrate multi-tiered responses to biotic stress. As computational pipelines and integrative analytical models continue to advance, multi-omics-driven breeding promises to redefine peanut improvement programs. By leveraging these technologies, breeders can dissect the genetic and biochemical basis of resistance with unprecedented precision and translate this knowledge into predictive selection models. Such integrative approaches will ultimately enable the development of peanut cultivars with durable, broad-spectrum resistance ensuring stable yield and food security in the face of intensifying biotic and climatic challenges [240].

### 8.1. Systems-Level Multi-Omics Insights into Foliar and Soil-Borne Disease Resistance in Peanut

Omics-based investigations have substantially advanced understanding of peanut resistance to plant pathogen by revealing how hormone-regulated and PAMP-triggered immune pathways are transcriptionally and metabolically orchestrated during pathogen challenge. RNA sequencing (RNA-seq) has emerged as a cornerstone technology for decoding the molecular architecture of peanut–pathogen interactions by enabling comprehensive, genome-wide dissection of defense pathways and regulatory hierarchies that govern disease resistance [241,242,243,244]. For example, comparative transcriptomic analyses of resistant and susceptible peanut genotypes infected with *Puccinia arachidis* demonstrate that resistance is underpinned by the targeted induction of defense-associated genes, including MLO-like proteins, ethylene-responsive factors (ERFs), F-box protein, and thaumatin. On the other hand, susceptible genotypes exhibit repression of key defense-related components such as pectin methyl-esterase inhibitor genes and lipoxygenase, which reflects impaired activation of lipid-mediated signalling and cell wall defense mechanisms [245]. These expression patterns underscore the importance of precise transcriptional control in determining host compatibility and disease outcome. Similar principles extend to leaf spot pathogens, where coordinated transcriptional activation of immune components underlies cultivar-specific disease tolerance [246,247].

Stem rot (caused by *Sclerotium rolfsii*) resistance in peanut likewise been illuminated through transcriptomics, revealing defense as a dynamic, multi-tiered signalling process rather than a static antimicrobial response. Resistant genotypes initiate a defense state dominated by salicylic acid-associated transcriptional programs, accompanied by structural reinforcement of host tissues through lignin deposition and oxidative enzyme activation. The coordinated induction of phenylalanine ammonia lyase, peroxidases, lignin polymerization, and β-1,3-glucanase reflects a shift toward durable, systemic resistance capable of constraining *S. rolfsii* colonization at both biochemical and physical levels [248]. RNA-seq-based analyses further captured temporal complexity, revealing dynamic modulation of both host immunity genes and fungal virulence factors, highlighting the bidirectional molecular dialogue that governs infection outcomes [249].

Mechanistic insights from integrative transcriptomics reveal that effective resistance is tightly coupled to early pathogen perception and signal amplification mechanisms characteristic of PAMP-triggered immunity. Resistant peanut genotypes show elevated expression of receptor-like kinases (RLKs) that act as primary surveillance components, followed by activation of jasmonic acid-responsive regulatory circuits. Downstream, WRKY transcription factors and zinc-finger proteins including C2-H2 domain containing regulators, operate as transcriptional hubs that coordinate large-scale defense gene reprogramming, enabling rapid and sustained immune deployment against stem rot [241]. Evidence accumulated across multiple peanut pathosystems converges on a unifying principle: effective resistance does not arise from the action of single resistance genes but is instead driven by large-scale, coordinated transcriptional reprogramming that synchronized pathogen perception, signal transduction, and defense execution. Comparative transcriptomic analysis between resistant and susceptible peanut genotypes have repeatedly exposed a conserved core of immune signalling architectures, prominently featuring mitogen-activated protein kinase (MAPK) cascades, plant–pathogen interaction pathways, and hormone-mediated defense networks. Within this regulatory landscape, WRKY transcription factors emerge as dominant control nodes, integrating early pathogen-derived signals with extensive downstream activation of defense-associated gene cohorts and amplifying immune outputs [250]. Further, insights from wild peanut ancestors (*Arachis duranensis* and *A. ipaensis*) demonstrate that these immune architectures are evolutionary conserved, highlighting their strategic value as breeding targets [241].

Complementing transcriptomic insights, metabolomic profiling captures real-time physiological adjustments, thereby revealing the biochemical foundation of stress perception, metabolic prioritization, and defense execution during pathogen invasion [251]. Pathogen-induced metabolic reprogramming is particularly evident during *Fusarium oxysporum* infection, which provokes a profound redistribution of cellular resources. Infection is typically accompanied by suppression of photosynthetic machinery alongside upregulation of energy metabolism, protein synthesis, and turnover, reflecting a strategic redirection of metabolic investment toward defense [252,253,254,255]. Concomitantly, core metabolic pathways involving glucose metabolism, cell wall biosynthesis, ROS regulation, pathogenesis-related (PR) proteins, and secondary metabolites, such as flavonoids and polyphenolics are extensively remodelled to reinforce structural barriers and antimicrobial capacity [256].

In peanut, these metabolic defense strategies are tightly linked to enzymatic and secondary metabolic reinforcement. Upon *F. oxysporum* infection, immune-related genes such as PAL and 4CL are strongly induced, promoting the synthesis of key enzymes including superoxide dismutase (SOD), phenylalanine ammonia-lyase (PAL), 4-coumarate-CoA ligase (4CL), and chalcone isomerase (CHI). This enzymatic activation drives the accumulation of defense-associated metabolites such as polyphenols, flavonoids, malondialdehyde (MDA), soluble sugars, total flavonoids (TF), and total phenolics (TP), collectively enhancing antioxidant capacity and reinforcing innate immunity [257]. Parallel evidence from other systems, such as castor bean, shows that upregulation of cinnamoyl-CoA reductase (CCR-1), NLR gene (e.g., RPP8), and chitinases contributes to lignin biosynthesis and ROS detoxification through elevated SOD activity, further illustrating conserved metabolic defense logic across plant species [258,259]. Together, transcriptomic and metabolomic evidence positions peanut disease resistance as a systems-level phenotype arising from integrated gene-metabolite networks. Shared mechanisms including ROS detoxification, secondary metabolite biosynthesis, structural reinforcement, and stress-responsive signalling operating across pathogen classes, while pathogen-specific transcriptional and metabolic adjustments fine-tune responses. This multi-omics perspective provides a robust framework for identifying biomarkers, building predictive resistance models, and implementing precision breeding to develop peanut cultivars with durable, broad-spectrum disease resistance.

### 8.2. Multi-Omics Regulation of Aspergillus flavus Resistance and Aflatoxin Defense Networks in Peanut

Resistance to *A. flavus* infection and aflatoxin contamination in peanut is a complex, multilayers trait that emerges from the coordinated interplay of biochemical, transcriptional, proteomic, and metabolic defense systems. Multi-omics investigations have demonstrated that fungal challenge triggers a cascade of tightly regulated responses rather than isolated defense events, with early infection stages characterized by pronounced changes in key defense enzyme activities, including phenylalanine ammonia lyase (PAL) [89], peroxisome (POD) and lipoxygenase (LOX) [260,261]. These enzymes, consistently detected across enzymatic assays, transcriptomic profiling, and proteomic analyses participate in lignin biosynthesis, phenolic accumulation, and oxylipin signalling, thereby reinforcing cell walls, maintaining redox balance, and limiting reactive oxygen species-mediated damage during fungal invasion [91].

Beyond general stress mitigation, several resistance-associated enzymes and metabolites exert direct antifungal and antiaflatoxigenic effects. LOX-driven fatty metabolism generates signalling molecules that activate downstream resistance pathways, promoting tolerance to both mechanical damage and pathogen attack [262]. At the regulatory level, WRKY transcription factors, TIR-NBS-LRR, heat shock proteins gene families function as central nodes within multi-omics-derived network governing *A. flavus* resistance [263]. Pathogenesis-related genes, including chitinase and PR10 proteins, are repeatedly identified across transcriptomic and proteomic datasets as critical contributors to fungal growth inhibition and aflatoxin suppression [94,264].

The integrative power of multi-omics is exemplified by combined transcriptomic and proteomic analyses of the resistant J11 peanut genotypes during *A. flavus* infection, which identified 663 differentially expressed genes and EGs and 314 differentially accumulated proteins, revealing extensive immune reprogramming at both transcriptional and translational levels [265]. Another integrative transcriptomic-metabolomic analyses have demonstrated that peanut responses to *A. flavus* infection converge predominantly on phenylpropanoid biosynthesis and linolenic acid metabolism pathways, providing mechanistic insight into how transcriptional regulation is reflected at the metabolic level. This study identified key resistance-associated metabolites, including Resveratrol, cinnamic acid, coumaric acid, ferulic acid, and 13S-HPODE, as major contributors to antifungal defense, underscoring the biochemical effectiveness of coordinated gene-metabolite responses during infection [266].

Proteomics-driven studies have further revealed that peanut defense networks can discriminate between toxigenic and non-toxigenic *A. flavus* strains. Differential proteomic profiling of peanut cotyledons infected with aflatoxin-producing strains identifies a suite of aflatoxin-responsive proteins involved in immune signalling, PAMP perception, penetration resistance, hypersensitive response, nucleic acid stabilization, phytoalexins and condensed tannin biosynthesis, cell wall remodelling, detoxification, and metabolic regulation, while these responses were absent in interactions with non-toxigenic strains [267]. These findings highlight a toxin-specific layer of immune regulation that is only apparent through integrative proteomic analyses.

Large-scale transcriptomic analysis of post-harvest peanut seeds from resistant and susceptible genotypes markedly advanced understanding of *A. flavus* resistance, revealing over 30,000 differentially expressed unigenes and 842 defense-related candidates. These genes including PR proteins, leucine-rich repeat receptor-like kinases, transcription factors, MAPKs, NBS–LRR proteins, polygalacturonase inhibitor proteins, and ADP-ribosylation factors which collectively delineate a complex immune regulatory network underlying resistance and reduced aflatoxin accumulation [268]. Network-based reanalysis of public RNA-seq datasets further reinforced these findings, revealing additional resistance-associated candidates, including R proteins, protein 21, pattern recognition receptors, thaumatin-like protein 1b, pectinesterases, and laccase [269], as well as MAPK kinase, serine/threonine kinase (STK), pattern recognition receptors (PRRs), pathogenesis related proteins (PR10), cytochrome P450, 1 aminocyclopropane-1carboxylate oxidase (ACO1), phosphatidylinositol transfer protein, SNARE protein SYP121, and pentatricopeptide repeat (PPR) proteins [270].

Environmental context further modulates these defense networks, as demonstrated by comparative proteomic profiling of resistant (YJ-1) and susceptible (Yueyou 7) peanut cultivars under combined drought stress and *A. flavus* infection. Differential accumulation of proteins such as PII proteins, oxidases, SAP-domain proteins, kinases, iso-Ara h3, peroxidase, trypsin inhibitors, and heat shock protein precursors [271]. Complementary transcriptomic-metabolomic studies show that resistant cultivars initiate faster and more coordinated responses, coupling activation of stress signalling, cell wall reinforcement, and secondary metabolism, while simultaneously accumulating protective metabolites. Such coordinated gene-metabolite interactions reveal regulatory networks that are central to effective resistance rather than isolated defense responses [266]. Metabolomics-driven analyses further refine this systems-level framework by revealing tissue-specific defense strategies, particularly within the seed coat, a critical physical and biochemical barrier to fungal invasion. Untargeted metabolomic profiling across cultivars with contrasting resistance levels identified pronounced differences in antioxidant capacity and antifungal activity driven by secondary metabolite composition. Phenylpropanoid- and flavonoid-derived compounds, including anthocyanins, aurones, and chalcones, showed strong positive associations with resistance, while integrated transcriptomic data linked these metabolic phenotypes to differential regulation of flavonoid and anthocyanin biosynthetic genes, directly connecting pigment metabolism to enhanced antioxidative defense and suppression of *A. flavus* growth [272]. Collectively, these studies establish peanut resistance to *A. flavus* and aflatoxin contamination as an emergent property of interconnected transcriptional, proteomic, and metabolic networks rather than single resistance determinants. The convergence of multi-omics data provides a powerful framework for biomarker discovery, resistance prediction, and data-driven precision breeding aimed at developing durable, aflatoxin-resilient peanut cultivars.

## 9. Challenges and Future Prospects

Despite remarkable progress in molecular genetics and breeding technologies, precision breeding in peanut still faces substantial biological, technical, and regulatory challenges. The allotetraploid nature of cultivated peanut (*Arachis hypogaea* L.; AABB genome) presents significant barriers to precise genome manipulation due to extensive sequence redundancy and homoeologous gene masking. These complexities make it difficult to accurately target and edit specific alleles controlling disease and pest resistance. Furthermore, low transformation efficiency and genotype-dependent tissue culture regeneration remain major bottlenecks in the application of genome editing tools. Although recent advances have produced more complete reference genomes, critical gaps persist in pan-genomic coverage, functional annotations, and marker–trait associations, limiting the identification and deployment of robust resistance genes against rapidly evolving pathogens and pests.

Regulatory and societal constraints further slow the translation of laboratory discoveries into field applications. In several regions, genome-edited crops are still regulated under transgenic frameworks, creating uncertainty and delaying approvals for field testing and commercialization. Farmer adoption also faces barriers related to biosafety perceptions, limited access to edited germplasm, and lack of awareness regarding the advantages of genome-edited cultivars. Therefore, harmonization of global biosafety policies and inclusive stakeholder engagement will be essential to ensure the equitable deployment of precision-bred peanut varieties.

A promising paradigm shift is emerging with the integration of remote sensing, machine learning (ML), and artificial intelligence (AI) into breeding programs. Advanced imaging technologies, such as multispectral, hyperspectral, and thermal sensors, enable early, non-destructive detection of disease and pest stress, providing high-throughput and objective phenotyping in natural field environments. Machine learning algorithms further strengthen these systems by analysing complex datasets that combine genomic, spectral, and environmental information to predict disease progression, quantify pest damage, and identify resistance-associated traits with high precision. This digital integration bridges the genotype–phenotype gap and enhances the efficiency of selection in precision breeding pipelines.

Looking forward, the future of peanut improvement lies in developing an integrated, data-driven framework that unites multi-omics, genome editing, phenomics, and AI-driven analytics. Such convergence will facilitate the discovery and precise modification of resistance gene networks, while real-time digital phenotyping will support adaptive selection under variable agro-climatic conditions. However, a major unknown persists: the field stability, epigenetic consistency, and long-term performance of edited resistance genes remain largely untested across diverse environments. Addressing these uncertainties alongside strengthening research capacity, regulatory reform, and digital infrastructure will be critical for realizing the full potential of precision breeding in creating climate-resilient, multi-stress-tolerant, and globally adoptable peanut cultivars.

## Data Availability

The data is within the manuscript. Further inquiries can be directed to corresponding authors.
